# The Role of NRF2/KEAP1 Signaling Pathway in Cancer Metabolism

**DOI:** 10.3390/ijms22094376

**Published:** 2021-04-22

**Authors:** Moon-Young Song, Da-Young Lee, Kyung-Soo Chun, Eun-Hee Kim

**Affiliations:** 1 College of Pharmacy and Institute of Pharmaceutical Sciences, CHA University, Seongnam 13488, Korea; wso219@naver.com (M.-Y.S.); angela8804@naver.com (D.-Y.L.); 2 College of Pharmacy, Keimyung University, Daegu 42601, Korea

**Keywords:** NRF2, KEAP1, cancer metabolism, metabolic reprogramming

## Abstract

The nuclear factor-erythroid 2 p45-related factor 2 (NRF2, also called *Nfe2l2*) and its cytoplasmic repressor, Kelch-like ECH-associated protein 1 (KEAP1), are major regulators of redox homeostasis controlling a multiple of genes for detoxification and cytoprotective enzymes. The NRF2/KEAP1 pathway is a fundamental signaling cascade responsible for the resistance of metabolic, oxidative stress, inflammation, and anticancer effects. Interestingly, a recent accumulation of evidence has indicated that NRF2 exhibits an aberrant activation in cancer. Evidence has shown that the NRF2/KEAP1 signaling pathway is associated with the proliferation of cancer cells and tumerigenesis through metabolic reprogramming. In this review, we provide an overview of the regulatory molecular mechanism of the NRF2/KEAP1 pathway against metabolic reprogramming in cancer, suggesting that the regulation of NRF2/KEAP1 axis might approach as a novel therapeutic strategy for cancers.

## 1. Introduction

Metabolic reprogramming is one of the characteristics of cancer [1,2]. Cancer metabolism plays an important role in tumorigenesis, which is strongly associated with pathways that regulate cell proliferation, stress response, genome stability, toxic responses, and bioenergetics [3]. These metabolic modifications not only increase key metabolic pathways such as glycolysis, pentose phosphate pathway (PPP), and glutaminolysis but also interact with multiple oncogenic signaling pathways such as phosphoinositide 3-kinase/protein kinase B- (PI3K/AKT), Ras-, p53-, Myc-, and reactive oxygen species (ROS)-related pathways [4,5,6,7,8,9]. One of the master regulators of cellular antioxidant response signaling, the nuclear factor erythroid 2-related factor 2 (NRF2)/Kelch-like-ECH-associated protein 1 (KEAP1) pathway affects several aspects of metabolic reprogramming. The NRF2/KEAP1 axis controls both the basal and stress-inducible expression and function of key metabolic components belonging to metabolic reactions such as glutathione biosynthesis and recycling, thioredoxin reductase, and thioredoxin signaling [10]. In addition, NRF2 controls key metabolic enzymes associated with the inhibition of adipogenesis, facilitation of flux through PPP, nicotinamide adenine dinucleotide phosphate (NADPH) regeneration, and increased purine biosynthesis [11,12,13,14,15,16,17,18,19,20]. It is noteworthy that the NRF2/KEAP1 signaling pathway has previously been shown to play a critical role in tumorigenesis and the correlation between redox and metabolism in cancer [12]. In this review, we summarize the components of the NRF2/KEAP1 pathway and the regulation of metabolic reprogramming by NRF2/KEAP1 axis. Furthermore, we discuss the potential strategies and therapeutic significance of NRF2/KEAP1 signaling pathway in cancer metabolism.

## 2. The Structure and Functions of NRF2/KEAP1

### 2.1. NRF2

**Figure 1:**
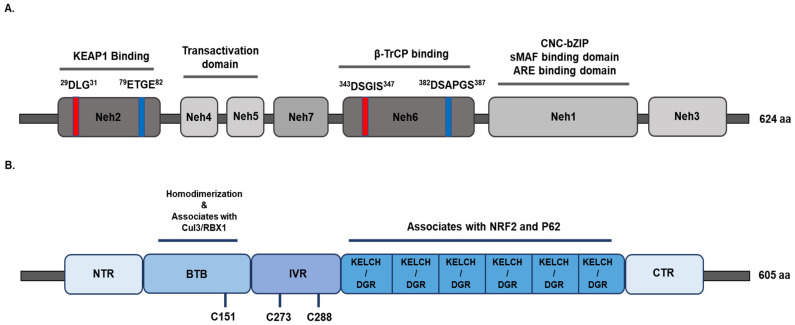
Domain structures of nuclear factor erythroid 2-related factor 2 (NRF2) and Kelch-like-ECH-associated protein 1 (KEAP1). (**A**) The relative positions of NRF2-ECH homology (Neh) domains, Neh1-Neh7, are indicated. The N-terminal Neh2 domain contains DLG and ETGE motifs, which interact with KEAP1. The Neh3, Neh4, and Neh5 domains are known as transactivation domains. The Neh6 domain contains the β-TrCP1 adaptor protein, which mediates proteasomal degradation. The c-terminal domain, Neh1, contains a CNC-bZIP, and it is responsible for heterodimerization with small MAF proteins (sMAFs). (**B**) KEAP1 consists of five domains that include the amino terminal region (NTR), a broad complex, tramtrack, bric-a-brac (BTB) domain, an intervening region (IVR), six Kelch domains, and the C-terminal region (CTR). The BTB domain is associated with CUL3-E3-ligase binding and the formation of keap1 homodimerization. The IVR contains several important cysteine residues that are responsible for modulating KEAP1-NRF2 activity. The Kelch/DGR domain is associated with NRF2 and P62, which is required for ETGE motifs. β-TrCP, β-transducin repeat-containing protein; CNC, cap‘n’collar; bZip, basic region leucine zipper; sMAFs, musculoaponeurotic fibrosarcoma protein; Cul3, Cullin 3.

The NRF2 signaling pathway has a critical role in regulating cellular and tissue homeostasis and protecting cells against the management of oxidative and electrophilic stress [21,22,23]. NRF2 is encoded by the gene *Nfe2l2* and belongs to a member of the Cap’n’collar (CNC) basic leucine zipper (bZIP) transcription factor family. It entails members having a conserved 43 amino acid homology region called the CNC, which contributes to the DNA-binding specificity of this family located at the N-terminal DNA-binding domain [24,25]. NRF2 is a modular protein and it consists of seven functional domains, known as the NRF2 ECH homology (Neh) domains Neh1–Neh7 [22,26,27,28]. As depicted in Figure 1A, the Neh1 domain contains a CNC-bZIP DNA-binding motif that allows NRF2 to dimerize with small Maf proteins and other transcription factors [25,27]. The Neh1 domain has also been reported to interact with UbcM2, an ubiquitin-conjugating enzyme, to regulate the stability of NRF2 [29]. The Neh2 domain is located in the N-terminus of NRF2, and it negatively controls the NRF2 through its DLG and ETGE motifs [30,31]. The two binding sites in Neh2, called DLG and ETGE motifs, help for NRF2 stability, and seven lysine residues are responsible for ubiquitin conjugation [30,32]. Importantly, the DLG and ETGE motifs bind with KEAP1, which is a substrate adaptor protein for the Cullin 3 (Cul3)-dependent E3 ubiquitin ligase complex that suppresses NRF2 by promoting its subsequent proteasomal degradation and ubiquitination [33,34,35]. The C-terminal Neh3 domain of NRF2 interacts with coactivators to facilitate the transactivation of NRF2 target genes. The Neh3 domain recruits chromo-ATPase/helicase DNA-binding protein family member CDH6, which functions as an NRF2 transcriptional coactivator [36]. The Neh4 and Neh5 domains are also important for the transactivation of NRF2 target genes and interact with cAMP response element-binding protein (CREB)-binding protein (CBP) and/or receptor-associated coactivator (RAC) [37,38]. The Neh6 domain negatively regulates NRF2 through DSGIS and DSAPGS motifs, β-transducin repeat-containing protein (β-TrCP). β-TrCP is a substrate adaptor for the S-phase kinase-associated protein 1 (SKP1)–Cul1–RING-box protein (Rbx1)/Roc1 ubiquitin ligase complex. DSGIS motif is phosphorylated by glycogen synthase kinase (GSK)-3β and increases the ability of β-TrCP to ubiquitinate NRF2 and promotes its rapid conversion [39,40,41]. The seventh Neh domain is known as Neh7 that mediates the repression of NRF2 interacting with the retinoic X receptor alpha (RXRα) and represses NRF2 target gene transcription [42].

### 2.2. KEAP1

KEAP1, a substrate adaptor for a Cul3-containing E3 ubiquitin ligase, interacts with NRF2 and controls the stability of NRF2. KEAP1 possesses five domains (Figure 1B), including N-terminal region (NTR), the Broad complex Tramtrack and Bric-à-Brac (BTB) domain, the intervening region (IVR), the Kelch domain/double glycine repeat (DGR), and the C-terminal region (CTR) [43]. The BTB domain binds with Cul3 and mediates KEAP1 homodimerization that is critical for ubiquitination and the proteasomal degradation of NRF2 [44,45]. Additionally, the BTB domain consists of cysteine residue Cys151 that is associated with oxidative stress level [46]. The IVR domain contains reactive cysteine residues such as Cys257, Cys273, Cys288, and Cys297 that have been proposed to promote KEAP1-dependent NRF2 ubiquitination [47]. The Kelch/DGR domain includes six Kelch repeats that interact with the binding of KEAP1 to the ETGE or DLG motifs located within the Neh2 domain of NRF2 [48,49,50]. Three functional domains of KEAP1 play critical roles in mediating NRF2 repression and ubiquitination.

### 2.3. Regulation of the Stability of NRF2/KEAP1 Complex

**Figure 2:**
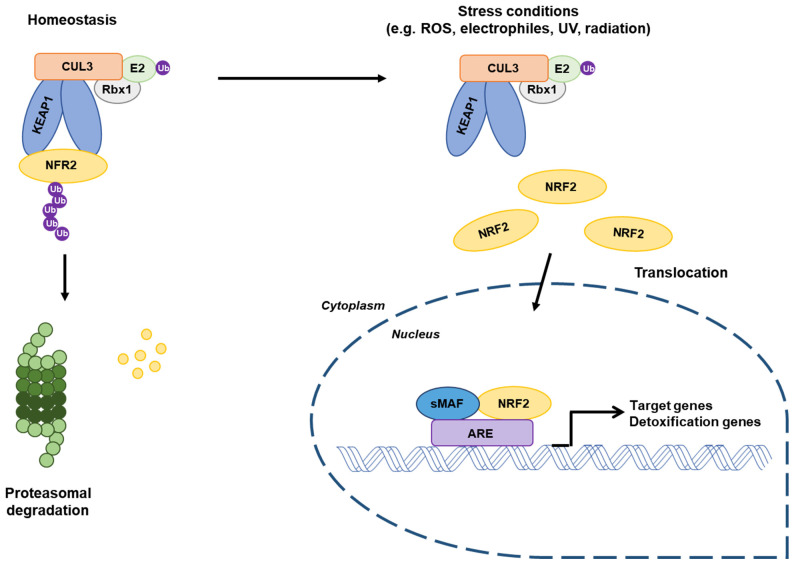
Regulation of NRF2 molecular mechanisms. Under basal conditions, NRF2 binds to its suppressor KEAP1 in the cytosol and interacts with the Cul3-RBX1 E3 ubiquitin ligase that constantly leads to NRF2 ubiquitination and proteasomal degradation. Under stressed conditions, conformational changes in KEAP1 and dissociation of NRF2 occur. Stabilized NRF2 translocates into the nucleus and forms a dimer with sMAFs proteins. The complex binds to antioxidant responsive elements sequences, promoting the transcription of target genes. RBX1, RING-box protein. Adapted from Jaramillo and Zhang [54].

KEAP1 is one of the major regulators of intracellular levels of NRF2. Under basal conditions, NRF2 is repressed by proteasomal degradation mediated by KEAP1. NRF2 is primarily localized in a cytosol and binds with KEAP1 as a dimer via the KEAP1 Kelch domain and ETGE/DLG motifs of NRF2 (Figure 1B), and it promotes NRF2 ubiquitination, leading to a subsequent proteolysis. Therefore, KEAP1 tightly regulates the expression of NRF2 to a low level in order to avoid the unnecessary expression of its target genes [30,51,52]. However, KEAP1 is exposed by ROS, electrophiles, or another stressor at major cysteine residues, leading to conformational change in the KEAP1/Cul3/RBX/NRF2 complex and loss of NRF2 ubiquitination [47,49]. As a consequence, NRF2 dissociates from KEAP1 and translocates into the nucleus, where it heterodimerizes with small Maf proteins (sMAFs) and subsequently binds to the antioxidant responsive elements (AREs) located in the promoter of NRF2 target genes (Figure 2) [28,51,52,53]. An additional signaling pathway for the KEAP1-independent regulation of non-canonical NRF2 stability has been revealed. The β-TrCP–SKP1–RBX1–CUL1 E3 ubiquitin ligase complex regulates NRF2 for proteasomal degradation upon GSK-3β-dependent phosphorylation within the Neh6 domain of NRF2 [40]. 

### 2.4. Functions of NRF2 as a Transcription Factor

NRF2 coordinately regulates coding for detoxification or metabolic enzymes, antioxidants, NADPH regeneration enzymes, and multi drug-metabolizing enzymes. NRF2 controls the redox status of glutathione (GSH) homeostasis by directly regulating two subunits of the glutamate–cysteine ligase catalytic (GCLC) and modifier (GCLM) subunits involved in glutathione biosynthesis [55,56]. Moreover, several ROS-detoxifying enzymes such as glutathione peroxidase 2 (GPX2), glutathione S-transferases (GSTA1,2,3,5, GSTM 1–3, and GSTP1) [57,58], but also a GSH-based antioxidant system, thioredoxin 1 [55], thioredoxin reductase 1 [59,60,61], and thioredoxin-inhibitor are transcriptionally regulated by NRF2 [62,63,64]. Furthermore, NRF2 plays an important role in xenobiotics and drug detoxification by controlling the expression of drug-metabolizing enzymes while requiring NADPH as a cofactor. The expression of NADPH-generating enzymes such as glucose-6-phosphate dehydrogenase (G6PD), 6-phosphogluconate dehydrogenase (PGD), isocitrate dehydrogenase 1 (IDH1), and malic enzyme 1 (ME1) are also regulated by NRF2 [18,20,65]. Together, NRF2 activity ensures the expression of enzymes against xenobiotics and oxidative stress as well as catalyzing reductive reactions. Evidence has shown that NRF2 regulates more than 200 genes involved in cellular processes, cytoprotection, metabolism, and gene transcription, as listed in Table 1.

## 3. NRF2 /KEAP1 Axis-Mediated Metabolic Reprogramming in Cancer

One of the classical pathways against metabolic reprogramming is the Warburg effect or aerobic glycolysis [70]. In normal cells, glycolysis is a physiological response to hypoxia. However, unlike normal cells, cancer cells constitutively increase glucose uptake and catabolize glucose into lactate regardless of oxygen availability [5,71]. Accelerating glycolytic flux allows producing ATP as well as fulfilling the metabolic demands of proliferating cells [70,71]. These metabolic properties of the cancer cells increase the synthesis of DNA and lipid [72]. Despite the aberrant activation of the cancers, it appears to involve a general induction against several pathways that support key functions such as redox balance, anabolic, and catabolic [73]. The NRF2/KEAP1 signaling pathway has been reported as a transcription factor that activates the antioxidant genes. However, more evidence reveals that the NRF2/KEAP1 signaling pathway is correlated with metabolic reprogramming in various cancer cells through several mechanisms [11,74]. It is becoming clear that the NRF2/KEAP1 pathway plays an important role in exerting the metabolic reprogramming of cancer cells through a transcriptional program inducing the proliferation of cancer cells and malignant progression. In addition, NRF2 supports intermediate metabolism through glutaminolysis [75], thereby generating an imbalance in metabolic processes such as the biosynthesis of amino acids [20] and nucleotides [17]. This section provides information on the role of the NRF2/KEAP1 axis in the regulation of the cancer metabolism against the cancer redox homeostasis and metabolic mechanisms.

### 3.1. The Role of NRF2/KEAP1 in Cellular Metabolism

#### 3.1.1. PI3K/AKT Signaling Pathway

NRF2 plays a critical role in the proliferation of cancer cells via metabolic reprogramming. When growth factors are stimulated in normal cells, PI3K signaling activation and its downstream AKT and mammalian rapamycin target (mTOR) promote programming including increased activity glycolytic flux as well as fatty acid synthesis [76]. However, in the oncogenic pathway, the PI3K/AKT pathway serves as a major proliferative signal by interacting with the NRF2 signaling. The PI3K signaling pathway has been reported to control the regulation of NRF2 signaling independently of KEAP1 [77,78,79]. When insulin-like growth factor (IGF) receptor is activated, PI3K catalyzes the phosphorylation of the lipid phosphatidylinositol 4,5-bisphophate (PIP2) to produce phosphatidylinositol 3,4,5-triphosphate (PIP3). The generation of PIP3 is important for the AKT activity, which mediates downstream signaling events including the inhibition of the GSK-3β [80,81]. GSK-3β is a key mediator that is inhibited by AKT-mediated phosphorylation [82], and NRF2 is phosphorylated by GSK-3β, enabling its recognition by β-TrCP that in turn marks NRF2 for ubiquitination regardless of the mediated by engaging the KEAP1/CUL3 complex [39,41,83]. When the GSK-3β is inactivated by phosphorylation, resulting in NRF2 accumulation through inhibition of KEAP1-independent degradation and increased abundance in NRF2, it promotes the activation of metabolic genes as well as anabolic metabolism, especially in the presence of active PI3K–AKT signaling [17]. Evidence shows that the PI3K pathway contributes to the activation of NRF2 in several contexts. The PI3K is antagonized by the tumor suppressor phosphatase and tensin homolog (PTEN) [84]. When the concentration of PTEN is low, AKT is activated, while GSK-3β is inhibited. Inhibition of GSK-3β has been reported to reduce NRF2 phosphorylation; subsequently, NRF2 escapes KEAP1-independent and β-TrCP-CUL1-dependent degradation in the nucleus. Thus, deletion of PTEN has been observed to increase the nuclear accumulation of NRF2 and the expression of NRF2 target gene [17]. Similarly, deletion of KEAP1 and PTEN leads to significantly increased NRF2 level in liver cells [85] and contributes to the tumorigenic potent on PTEN-deleted prostate cancer cells [86]. Additionally, inhibition of the PI3K/AKT pathway markedly reduces endogenous NRF2 protein and enzymes in *KEAP1*-mutant lung cancer cells and *KEAP1*-deficient mouse embryonic fibroblasts (MEFs) [40]. Taken together, activation of the PI3K/AKT signaling pathway increases the nuclear translocation of NRF2, independent of KEAP1, and allows NRF2 to promote metabolic reprogramming and increase cell proliferation.

#### 3.1.2. p62

Previous studies have demonstrated that NRF2 and p62/Sequestosome 1 protein (p62/SQSTM) can regulate the activity of NRF2 [83,87,88]. p62/SQSTM1 is a scaffold protein that regulates selective cytoplasmic aggregators of ubiquitinated proteins and organelles for degradation through the autophagy pathway. p62 contains an STGE motif that is similar to the NRF2 ETGE motif; it directly interacts with the Kelch domain of KEAP1 and the accumulation of p62 triggers serine phosphorylation of an STGE motif [83,88]. This phosphorylation enhances p62/KEAP1 interaction, aggregation, and autophagic degradation, which results in the stabilization of NRF2. However, this adaptive response can become pathological under circumstances of impaired autophagy, thereby triggering the accumulation of cytoplasmic protein inclusions in a p62- and NRF2-dependent manner [89,90,91]. The phosphorylation of p62 at S349 activates NRF2 and directly targets not only glucose metabolism to the glucuronate pathway but also glutamine metabolism to glutathione synthesis [92]. This is relevant, given that the amplification of the p62 gene and aberrant accumulation of phosphorylated p62 protein have been implicated in the acceleration of cancer development. In hepatocellular carcinoma cells, these changes confer the resistance against anti-cancer drugs and trigger cell proliferation. Furthermore, phosphorylated p62 has been reported to accumulate in tumor regions positive for hepatitis C virus [93].

#### 3.1.3. AMP-Activated Protein Kinase

AMP-activated protein kinase (AMPK) is a master regulator of metabolism and energy homeostasis in normal cells. In times of energy deprivation, the levels of ATP are decreased, and NRF2 deficiency may lead to AMPK activation. AMPK activation has been known to lead to the upregulation of glucose, fatty acid uptake, activation of autophagy, and an increase in ATP levels [89]. Several studies report that the AMPK promotes the activation of NRF2 [94], and a recent study revealed that the AMPK phosphorylates on serine 588 of NRF2 [95]. The activation of AMPK regulates the phosphorylation and inhibition of GSK-3β [90], which promotes the degradation of NRF2 through β-TRCP/CUL1 pathway. Therefore, AMPK regulates the nuclear localization and stabilization of NRF2.

#### 3.1.4. Crosstalk between Mitochondrial Metabolism

Mitochondria are major organelles responsible for ATP synthesis and cellular processes. In addition, it controls tricarboxylic acid (TCA) cycle, calcium, and ROS homeostasis, fatty acids, and amino acids metabolism. It is noteworthy that the mitochondrial dysfunction is caused by metabolic rewiring [91]. The NRF2-dependent interactions in metabolism and homeostasis affect mitochondrial function. Recently, it has been shown that NRF2 is activated by aberrant accumulation of the TCA cycle. In the absence of NRF2, glucose oxidation and the flowing of substrate into the TCA cycle are diminished [91]. In contrast, the constitutive activation of NRF2 has been observed to induce the glucose oxidation and flowing of substrate into the TCA cycle [96]. ATP levels were also observed to be decreased in *NRF2*-deficient MEFs, and on the contrary, the constitutive activation of NRF2 increased the levels of ATP [97]. Similarly, the silencing of NRF2 caused the decrease of ATP production and oxygen consumption in human colon cancer cells [98]. In particular, inactivation of fumarate hydratase (FH) triggers the accumulation of the TCA cycle intermediates, fumarate and succinate, which lead to an interruption in KEAP1/NRF2 binding [99,100]. Fumarate has been reported to interact with cysteine residues within the KEAP1 protein upon the activation of NRF2 [99,100]. Furthermore, a loss of FH locus has been shown to lead to an aggressive form of renal cancer in hereditary leiomyomatosis and renal cell carcinoma patients [100,101,102,103]. Collectively, these findings suggest that the NRF2 interacts dependently with the axis of cancer metabolism and mitochondrial function. In addition, KEAP1 was found to be associated with mitochondrial interfaces. Interestingly, it has been revealed that KEAP1 exists in close proximity to the mitochondria and interacts with the mitochondrial outer membrane histidine phosphatase, PGAM5 [104,105,106]. The depletion of PGAM5 or NRF2 causes an inhibition of mitochondrial retrograde trafficking, due to activation of the KEAP1–cullin-3 E3 ubiquitin complex and rescued degradation of Miro2, which is a mitochondrial GTPase that links mitochondria to microtubules [104,105]. Furthermore, the PGAM5–KEAP1 complex induces oxeiptosis, a caspase-independent cell death program, under high ROS generated [107]. Whereas, under the unstressed conditions, KEAP1 is important role for the maintenance of mitochondrial homeostasis with p62 and Rbx1 through mitochondrial ubiquitination in liver disease [108].

### 3.2. Modulation of Metabolic Processes by NRF2/KEAP1 Signaling

#### 3.2.1. Pentose Phosphate Pathway

Glucose is an important source of cellular energy. It enters the cells through glucose transporters, and its metabolites serve as substrates for biosynthetic processes. Moreover, glucose provides other metabolic intermediates for biosynthetic pathways, such as PPP. The pentose phosphate pathway (also called the phosphogluconate pathway and the hexose monophosphate shunt) consists of the oxidative branch and the non-oxidative branch [109]. All the branches need the ribose-5-phosphate and NADPH for the changing demands of the cells. In the oxidative phase of PPP, the G6PD and PGD catalyze the reaction. Entry into the oxidative arm is catalyzed by G6PD, and G6PD has been known to be controlled by NRF2 [17]. It determines the flux of glucose through PPP and biosynthetic reactions. In another branch, the non-oxidative branch of PPP, NRF2 positively regulates the expression of transaldolase 1 (TALDO1) and transketolase (TKT) [17,19]. NRF2 plays a crucial role in promoting the proliferation of cancer cells and metabolism process in lung cancer cells, including the direct transcriptional regulation of PPP-related enzymes such as G6PD, PGD, TKT, and TALDO1, which are responsible for NADPH regeneration [17]. The G6PD, PGD, TKT, and TALDO1 support glucose flux and generate purines, which are building blocks of DNA and RNA, which help to accelerate proliferation in cancer cells. The ribose-5-phosphate is a major product of the PPP, and it is essential for the biosynthesis of nucleotides. The ribose-5-phosphate contributes the sugar group to nucleotides and ultimately forms the sugar backbone in DNA. The ribose-5-phosphate is metabolized to phosphoribosyl–pyrophosphate, which is a common precursor for both purine and pyrimidine nucleotides. Mitsuishi et al. has reported that NRF2 enhances the expression of purine base synthesis by the indirect regulation of phosphoribosyl–pyrophosphate amidotransferase, which catalyzes the rate-limiting step in the de novo purine biosynthesis, and methylenetetrahydrofolate dehydrogenase 2, which is a nuclear-encoded mitochondrial bifunctional enzyme with methylenetetrahydrofolate dehydrogenase and methenyltetrahydrofolate cyclohydrolase activities, thus initiating the creation of the purine ring [17]. Indeed, the biosynthesis of purine has been shown to be affected by the activity of NRF2, and it has been observed to flow continuously through the PPP. Moreover, carbon flux is increased in *KEAP1*-knockout MEFs and decreased in their *NRF2*-knockout counterparts.

#### 3.2.2. Amino Acid Metabolism

Accumulating evidence shows that NRF2 regulates the intracellular pool of amino acids by coordinating several molecular pathways such as biosynthesis, absorption, proliferation, cancer metabolic reprogramming, and redox balance. NRF2 influences not only metabolism but also intracellular concentrations of the cysteine/glutamate transporter system. Several studies have focused on the interaction between NRF2 and xCT, which is a transmembrane antiporter coded by the *SLC7A11* gene, which is increased in several cancers and has been known to mediate the extrusion of glutamate and support the redox homeostasis [110,111,112]. NRF2 was found to increase not only the expression of the *SLC7A11* but also the activity of xCT in breast cancer cells [110]. In contrast, silenced NRF2 suppressed both xCT and glutamate export in breast cancer cells [112]. Moreover, the correlation of NRF2 and *SLC7A11* has been revealed from the result in almost 950 cancer cell lines [113,114]. Indeed, glucose starvation induced the overexpression of *SLC7A11* and subsequent upregulation of glucose dependence for cell survival through the NRF2- and activating transcription factor 4 (ATF4)-dependent transcription in renal cancer cells [110]. ATF4 is a transcription factor that plays an important role in amino acid deprivation, metabolic stress, and ER stress. ATF4 targeted by NRF2 has been known to induce the expression of xCT promoter [115]. In a separate study, NRF2 controlled the transcription of key enzymes through ATF4 activation, which is involved in serine/glycine biosynthesis in non-small-cell lung carcinoma cells [116]. Indeed, this study shows that the activation of NRF2 and ATF4 is associated with the poorer prognosis in the lung cancer patients [116]. Moreover, it is revealed that the KRAS-dependent regulation of the ATF4 mechanism via PI3K/AKT signaling pathway required the activation of NRF2, suggesting that the activation of these axis mechanisms is related to the expression of amino acid transporters such as *SLC1A5*, *SLC38A2*, *SLC7A5*, *SLC7A1*, and *SLC7A11* genes [117]. In agreement with this, NRF2 can transcriptionally promote the expression of ATF4 and the coding for amino acid transporters (AATs) involved in the import of proline, tryptophan, alanine, glycine, and glutamine in the colon cancer cells [118]. Furthermore, it is worthy that the inhibition of AATs may trigger the apoptosis in autophagy-deficient colorectal cancer cells but not wild-type colorectal cancer cells upon glutamine withdrawal [118].

In additionally, KEAP1 is involved in cysteine/glutamate metabolism. When metabolic and redox response pathways are activated, KEAP1 requires significant energy as well as metabolic substrates such as carbon and sulfur, leading to depletion of the TCA cycle intermediates [111,119,120,121,122]. Moreover, the loss of the functional mutant-KEAP1 increased the dependence on glutamine in human KRAS-driven lung adenoma cell lines. KEAP1-mutant cells reduced the intracellular glutamate pool by increasing glutamate consumption for GSH synthesis and exporting glutamate via anti-porter xCT in exchange for cysteine [75,118]. These studies indicate that the NRF2/KEAP1 signaling pathway could regulate amino acid metabolism in malignant tumors.

#### 3.2.3. Lipid Metabolism

As for lipid metabolism, NRF2 positively regulates catabolic metabolism that is involved in the degradation of phospholipids and triglycerides, and enzymes involved in fatty acids oxidation. NRF2 has been reported to control the efficiency of fatty acids oxidation by regulating the expression of the carnitine palmitoyltransferase isoforms (CPT1 and CPT2) within mitochondria and two peroxisomal enzymes, acyl-CoA oxidase 1 and 2 (ACOX1 and ACOX2), which are related in lipids beta-oxidation [16]. In *KEAP1*-wild-type and/or -knockout MEFs, the acceleration of fatty acid synthesis not only increased ATP production but also stimulated respiration [16]. Moreover, the absence of NRF2 has been reported to significantly decrease the efficiency of fatty acids oxidation [16]. Conversely, NRF2 has been shown to suppress the anabolic processes associated with lipid biosynthesis, fatty acid desaturation, and fatty acid transport [12,123]. These findings suggest that NRF2 may negatively regulate lipid biosynthesis and reduce the consumption of NADPH in cancer cells. In the murine models, hepatic mRNA levels of ATP-citrate lyase, acetyl-CoA carboxylase 1, fatty acid synthase, stearoyl CoA desaturase 1, and fatty acid elongase were downregulated by the activation of NRF2 and contrary suppressed in *NRF2*-knockout mice [16,19]. NRF2 transcriptionally regulates fatty acids oxidation-related genes and activates the degradation of damaged lipids, thereby reducing the form of NADPH in cancer cells. Taken together, these studies demonstrate that the NRF2/KEAP1 signaling pathway can regulate lipid metabolism such as lipid biosynthesis and fatty acid oxidation, respectively.

#### 3.2.4. Iron Metabolism

NRF2 is well known to play a key role in iron homeostasis. NRF2 controls the intracellular levels of the HO-1 enzyme and the storage of iron through the regulation of ferritin. Notably, NRF2 regulates the synthesis of heme, which is metabolized from heme to iron and biliverdin. NRF2 controls the expression of biliverdin reductase that metabolizes biliverdin to bilirubin and ferrochelatase, which is excreted as waste [19,124]. On the other side, heme biosynthesis needs the amino acid glycine through the NRF2–ATF4 serine biosynthesis pathway [20]. Accelerated iron synthesis during cancer development can induce carcinogenesis, cancer progression, and metastasis formation. The intracellular levels of the iron exporter ferroportin have been reported to be markedly downregulated in breast cancer cells, which is associated with accelerated cancer progression [125]. Furthermore, NRF2 has been reported to transcriptionally regulate proteins involved in iron and heme metabolism such as ferritin, ferrochlatase, heme-responsive gene 1, ferroportin, etc. [126]. This evidence suggests that NRF2 might play an important role in the cytoprotection and metabolic regulation through iron metabolism.

## 4. Conclusions

The NRF2/KEAP1 axis plays a major role in the cellular regulation of redox homeostasis, mitochondrial physiology, autophagy, proteostasis, immune system, and metabolism. The NRF2/KEAP1 complex is mediated by activating stimulation, interaction with other transcription factors, activators or repressors, and crosstalk with other signaling pathways. At the center of a complex regulatory network, the NRF2/KEAP1 pathway is emerging as a critical regulator of metabolism in cancer cells as its interactions with the metabolism-related pathway including the PI3K/AKT/mTOR pathway, p62 pathway, AMPK, and TCA cycle have been revealed. In addition, the NRF2/KEAP1 axis contributes to the several metabolic processes in cancers and the production of metabolites that promote cell proliferation and survival. In particular, the constitutive overexpression of NRF2 accelerates the proliferation of cancer cells, which is the result of the reprogramming of intracellular anabolic and catabolic metabolism. Understanding more integrated NRF2/KEAP1-mediated cancer metabolism may facilitate the discovery of new anti-cancer treatment strategies through cancer metabolic reprogramming.

## Figures and Tables

**Table 1:** A list of genes regulated by NRF2.

General Biochemical Function	Gene Symbol	Name	Refs
Biotransformation and detoxification (Phase I, II, III)	*ABCB6*	ATP-binding cassette, subfamily B (MDR/Tap) member 6	[59]
	*ABCC1*	ATP-binding cassette, subfamily C (CFTR/MRP)	[59]
	*ADH7*	Alcohol dehydrogenase class 4 mu/ sigma chain	[59]
	*CBR1*	Carbonyl reductase 1	[60]
	*CYP1B1*	Cytochrome P450	[59]
	*EPHX1*	Epoxide hydrolase 1, microsomal	[59,60]
	*UGT1A1*	UDP Glucuronosyltransferase 1	[60]
Anti-oxidant	*GCLC*	Glutamate–cysteine ligase, catalytic subunit	[66]
	*GCLM*	Glutamate–cysteine ligase, modifier subunit	[66]
	*GPX1*	Glutathione peroxidase 1	[60]
	*GSR1*	Glutathione reductase 1	[60]
	*PRDX1*	Peroxiredoxin 1	[59]
	*SRXN1*	Sulfiredoxin 1	[59]
	*TXN1*	Thioredoxin	[59]
Carbohydrate metabolism and NADPH generation	*G6PD*	Glucose-6-phosphate dehydrogenase	[61,62,67]
	*HDK1*	Hexokinase domain containing 1	[60]
	*IDH1*	NADP-dependent isocitrate dehydrogenase	[59]
	*ME1*	Malic enzyme 1	[66]
	*PGD*	6-phosphogluconate dehydrogenase	[66]
	*TALDO1*	Transaldolase	[60]
	*TKT*	Transketolase isoform 1	[60]
Lipid metabolism	*ACOT7*	Acetyl-CoA thioesterase 7	[19]
	*ACOX1*	Acetyl-CoA oxidase 1	[19]
	*SCD2*	Stearoyl-CoA desaturase-2	[19]
Heme and iron metabolism	*BLVRA*	Biliverdin reductase A	[60]
	*BLVRB*	Biliverdin reductase B	[60]
	*FTH1*	Ferritin, heavy polypeptide	[60]
	*FTL1*	Ferritin, light polypeptide	[60]
	*HMOX1*	Heme oxygenase 1	[60]
Proteasomal degradation	*ATF4*	Activating transcription factor-4	[68]
	*PSMA1*	Proteasome subunit alpha type-1	[68]
	*PSMB5*	Proteasome subunit beta type-5	[68]
	*SQSTM1*	Sequestosome 1 (p62)	[65]
Autophagy	*ATG5*	Autophagy protein 5	[59]
	*ATG7*	Autophagy protein 7	[59]
	*LC3B*	Microtubule-associated protein 1A/1B-light chain 3B	[67]
Apoptosis	*BCL2*	B-cell lymphoma 2	[69]

## Abbreviations

AATs	Amino acid transporters
ACOX	Acyl-CoA oxidase
AKT	Protein kinase B
AMPK	AMP-activated protein kinase
ARE	Antioxidant responsive element
ATF4	Activating transcription factor 4
BTB	Broad complex, tramtrack, bric-a-brac
bZIP	Basic leucine zipper
CBP	CREB-binding protein
CNC	Cap‘n’collar
CPT	Carnitine palmitoyltransferase isoforms
CREB	cAMP response element binding protein
CTR	C-terminal region
Cul3	Cullin 3
DGR	Double glycine repeat
FH	Fumarate hydratase
G6PD	Glucose-6- phosphate dehydrogenase
GCL	Glutamate–cysteine ligase
GPX	Glutathione peroxidase
GSH	Glutathione
GSK	Glycogen synthase kinase
GST	Glutathione S-transferases
IGF	Insulin-like growth factor
IVR	intervening region
KEAP1	Kelch-like-ECH-associated protein 1
MEF	Mouse embryonic fibroblast
NADPH	Nicotinamide adenine dinucleotide phosphate
NRF2	Nuclear factor erythroid 2-related factor 2
NTR	Amino terminal region
p62/SQSTM	p62/Sequestosome 1 protein
PGD	Phosphogluconate dehydrogenase
PI3K	Phosphoinositide 3-kinase
PIP2	Lipid phosphatidylinositol 4,5-bisphophate
PIP3	Phosphatidylinositol 3,4,5-triphosphate
PPP	Pentose phosphate pathway
PTEN	Phosphatase and tensin homolog
RAC	Receptor-associated coactivator
RBX1	RING-box protein
ROS	Reactive oxygen species
RXRα	Retinoic X receptor alpha
SKP1	S-phase kinase-associated protein 1
sMAF	Small musculoaponeurotic fibrosarcoma protein
TALDO1	Transaldolase 1
TCA	Tricarboxylic acid
TKT	Transketolase
β-TrCP	β-transducin repeat-containing protein
